# Assessing the Efficacy and Resident Satisfaction of Cardiac Surgery Training Programs in Saudi Arabia

**DOI:** 10.7759/cureus.51399

**Published:** 2023-12-31

**Authors:** Maryam A Alomair, Zainab N AlAithan, Waad Alotaibi, Abdulhakim I Alabdullah, Fay S Alhamad, Abdulmalek W Alhithlool

**Affiliations:** 1 Surgery Department, College of Medicine, King Faisal University, Alahsa, SAU; 2 College of Medicine, King Faisal University, Alahsa, SAU

**Keywords:** saudi arabia, satisfaction, structure, residency program, cardiac surgery

## Abstract

Introduction: The Saudi Cardiac Surgery Training Program, a recent addition to Saudi Arabia's medical landscape, demands proactive assessment for potential renovations. This study aims to assess the Cardiac Surgery Training Program's structure, utilizing stakeholder satisfaction as a predictive measure in Saudi Arabia.

Method: A cross-sectional study from March to September 2023 targeted current cardiac surgery residents in Saudi Arabia.

Result: Achieving a 76.4% response rate from 26 out of 34 residents. 65.4% of participants aged 25-30, 65.4% males, and 53.8% females were married. Financially, 46.2% earn less than 20,000 Saudi Riyals. Rotation effectiveness ratings highlighted strengths in cardiac surgery, thoracic surgery, and vascular surgery, with areas for improvement in mid-rotation feedback and exit interviews. Participants reported moderate constructive feedback use and occasional program ambiguity (both with a median of 3.00). Concerns about the future encompassed knowledge (34.6%), surgical skills (26.9%), and employability (26.9%) post-graduation. Satisfaction analysis revealed emotional exhaustion and frustration (both with a median of 4.00), contrasting with effective problem-solving skills (median of 7.00) and a positive influence on others' lives (median of 6.00). Educational satisfaction was high, with faculty care and a sense of being respected (both with a median of 3.00). Many individuals have expressed the intent to choose the program again, contributing to a moderate overall satisfaction level (median of 4.3).

Conclusion: These findings offer valuable insights for program enhancement, focusing on successes and addressing concerns to elevate the training experience and satisfaction of cardiac surgery residents in Saudi Arabia.

## Introduction

The Cardiac Surgery Training Program, overseen by the Saudi Commission for Health Specialties (SCFHS), represents a pivotal initiative in Saudi Arabia's medical education landscape. Regulated by the SaudiMED-CS 2020 competency framework, this program is the conduit for cardiac surgeons seeking continued specialization within the country [1]. Residency, a post-medical college phase, immerses participants in diverse inpatient and outpatient settings, handling elective and emergency cases involving both adult and pediatric patients.

As of 2021, 36 residents were reported to be enrolled in the program across 11 certified cardiac surgery training centers in Saudi Arabia [2]. The seven-year Saudi Board cardiac surgery program comprises two phases: a five-year junior level and a two-year senior level. The junior phase spans 156 weeks of general cardiac surgery and 104 weeks of advanced specialty cardiac surgery. Each phase integrates specific objectives and outcomes. Evaluation instruments encompass formative and summative methods and the shape of the curriculum. These include virtual session attendance, weekly quizzes, certifications in advanced cardiovascular life support (ACLS), basic life support (BLS), and 5C (COVID-19 Critical Care Crash Course), research involvement, presentations, a surgical procedures logbook, DOPS (direct observation for procedural skills), ITER/FITER (in-training evaluation and final report), and the Saudi Board Examination Parts 1 and 2, involving written and clinical assessments [3].

This study aims to evaluate the effectiveness of cardiac surgery training programs in Saudi Arabia, assess resident satisfaction, and evaluate the structural quality of cardiac surgery training programs using resident satisfaction as a success metric. While previous assessments have emphasized resident satisfaction in medical education, none have comprehensively explored its role in appraising the cardiac surgery training program's structural quality [4]. Resident satisfaction stands as a leading factor and a robust indicator of training program success [5]. This study endeavors to assess the Cardiac Surgery Training Program's structure, utilizing stakeholder satisfaction as a predictive measure in Saudi Arabia [6]. By shedding light on the need for improvements in surgical training, the findings may catalyze further research on resident satisfaction in other Saudi Arabian training programs.

## Materials and methods

Study design and setting

A cross-sectional study that was ethically approved by the Deanship of Scientific Research at King Faisal University in Saudi Arabia. Project No. KFU-REC-2023-FEB-ETHICS575. The cardiac surgery training program in Saudi Arabia is under the surveillance of the SCHS, which provides 11 centers for cardiac surgery distributed in the Saudi Arabian regions.

Study population

All residents of the Cardiac Surgery Saudi Board affiliated with SCHS (n=34) were eligible to participate in this study. All other residents from other specialties were excluded.

To enhance participation, the questionnaire was disseminated among cardiac surgery residents via WhatsApp and email. It is important to point out that the survey was voluntary and anonymous, and that the data were collected electronically and managed securely using Excel files. Twenty-six of the 34 residents in the cardiac surgery training program - including junior and senior levels - replied to the questionnaire, sharing their opinions on how satisfied they were with the curriculum.

Survey instrument

The initial questionnaire was meticulously organized into three well-defined sections. The first section focused on demographics, finances, and personal characteristics. The second section delved into comprehensive inquiries concerning the program structure. Finally, the third section explored satisfaction and work-life balance through multiple thoughtfully crafted questions.

Then the questionnaire underwent essential modifications to be aligned with the research objectives. The objectivity of the questionnaire was ensured by these modifications, which were suggested and approved by experts in the field of cardiac surgery. Google Forms was used to design and administer the survey instrument. It started with an introduction outlining the goal of the study and included informed consent at the beginning.

Statistical analysis

The Statistical Package for Social Sciences (SPSS, IBM Corp., Armonk, NY) computer program version 26, which is used for quantitative statistical analysis, was employed after the data had been first exported to Microsoft Excel (Microsoft® Corp., Redmond, WA). Descriptive statistics were used for each variable. Frequencies and percentages were calculated for categorical variables, and median and interquartile ranges for quantitative variables. Additionally, the reliability of the questionnaire was checked using Cronbach’s alpha and the result showed that the value of Cronbach’s alpha was 0.856, which implies that the questionnaire is highly reliable.

## Results

Demographics

Table 1 presents the demographic data of the participants. In terms of the participants' current residency year, the distribution is fairly balanced, with the majority of respondents being in either their first (19.2%), second (23.1%), or fifth (19.2%) year. Regarding the training location, the Central Region (Riyadh) emerges as the predominant location for training, accounting for 53.8% of respondents. The Western Region and Eastern Region each make up 23.1% of the participants. Age distribution reveals that a majority of respondents are between 25 and 30 years old (65.4%), with a smaller portion falling within the 30- to 35-year-old range (34.6%). The gender distribution indicates a higher representation of males (65.4%) compared to females (34.6%) among the participants. This could reflect broader gender demographics within the field of cardiac surgery in Saudi Arabia. The marital status of respondents shows an almost even split between single (46.2%) and married (53.8%) participants.

**Table 1:** Sociodemographic data. SA: strongly agree, A: agree, N: neutral, D: disagree, SD: strongly disagree.

Sociodemographic data		n	%
What is your current residency year?	First	5	19.2%
	Second	6	23.1%
	Third	2	7.7%
	Fourth	3	11.5%
	Fifth	5	19.2%
	Sixth	5	19.2%
	Seventh	0	0.0%
Where did you get most of the training?	Central Region (Riyadh)	14	53.8%
	Western Region	6	23.1%
	Eastern	6	23.1%
Age	25–30 years	17	65.4%
	30–35 years	9	34.6%
Gender	Male	17	65.4%
	Female	9	34.6%
Marital status	Single	12	46.2%
	Married	14	53.8%
If you are married, does your spouse also work in the healthcare field?	Yes	4	22.2%
	No	14	77.8%
Number of kids	0	16	61.5%
	1	8	30.8%
	2	1	3.8%
	More than 2	1	3.8%
Do you take care of others, like parents?	Yes	10	38.5%
	No	16	61.5%
If yes; financial only or financial + social	Financial only	2	18.2%
	Financial and social	9	81.8%
I would describe myself as a perfectionist	SD	0	0.0%
	D	1	3.8%
	N	13	50.0%
	A	10	38.5%
	SA	2	7.7%
I would describe myself as very empathetic	SD	0	0.0%
	D	0	0.0%
	N	7	26.9%
	A	14	53.8%
	SA	5	19.2%
I would describe myself as indecisive	SD	3	11.5%
	D	7	26.9%
	N	11	42.3%
	A	3	11.5%
	SA	2	7.7%
I would describe myself as idealistic	SD	1	3.8%
	D	2	7.7%
	N	9	34.6%
	A	11	42.3%
	SA	3	11.5%
My monthly salary is (Saudi Riyal)	Less than 20,000	12	46.2%
	20,000–30,000	13	50.0%
	30,000–40,000	0	0.0%
	More than 40,000	1	3.8%
My monthly salary is enough for essential requirements like food, accommodation, transportation, …etc.	SD	1	3.8%
	D	2	7.7%
	N	5	19.2%
	A	6	23.1%
	SA	12	46.2%

Family responsibilities, in terms of caring for others, reveal that a significant majority of participants do not have caregiving responsibilities (61.5%), while 38.5% do take care of others, potentially indicating the challenges and commitments that some trainees might face outside of their medical training. In terms of salary, the distribution shows that a considerable portion of participants earn less than 20,000 Saudi Riyals (46.2%), while an equal number earn between 20,000 and 30,000 (50.0%). Only a small portion earns more than 40,000 (3.8%), and no respondents fall within the 30,000-40,000 range.

Finally, respondents were asked about the perceived adequacy of their monthly salary for essential requirements. Notably, a significant portion of participants (46.2%) strongly agree (SA) that their salary is sufficient for essentials, while 23.1% agree (A), and 19.2% are neutral (N). This suggests a generally positive sentiment regarding salary adequacy.

Program data rotations effectiveness

In Table 2, participants provided evaluations of the effectiveness of various core rotations within the program, employing a rating system ranging from "Poor" to "Outstanding". Each rating was assigned a numeric value for analysis, with "Poor" assigned the lowest value (1) and "Outstanding" assigned the highest value (6). In the table, the "median rating" represents the central value of the ratings given by participants for a specific rotation. The "IQR" stands for Interquartile Range, a measure of the spread or dispersion of the ratings. It tells us how spread out the ratings are and provides insights into the variability of opinions among participants.

**Table 2:** Participants' evaluations of the effectiveness of various core rotations within the program.

Please rate the effectiveness of each of your core rotations	Poor	Acceptable	Good	Very good	Excellent	Outstanding	Median	IQR	Rank
Cardiac surgery	3	3	4	4	6	6	4.00	2.50	7
	11.5%	11.5%	15.4%	15.4%	23.1%	23.1%			
General surgery	4	9	5	4	2	0	2.00	1.75	13
	16.7%	37.5%	20.8%	16.7%	8.3%	0.0%			
Research	2	4	5	6	3	0	3.00	2.00	12
	10.0%	20.0%	25.0%	30.0%	15.0%	0.0%			
ICU	0	3	2	9	6	1	4.00	1.50	9
	0.0%	14.3%	9.5%	42.9%	28.6%	4.8%			
Thoracic surgery	2	1	1	3	7	2	5.00	1.75	2
	12.5%	6.3%	6.3%	18.8%	43.8%	12.5%			
Vascular surgery	0	1	0	4	11	3	5.00	1.00	3
	0.0%	5.3%	0.0%	21.1%	57.9%	15.8%			
Trauma	1	4	3	4	7	1	4.00	2.75	6
	5.0%	20.0%	15.0%	20.0%	35.0%	5.0%			
Cardiology-CCU	0	2	6	8	3	1	4.00	1.00	10
	0.0%	10.0%	30.0%	40.0%	15.0%	5.0%			
Cath lab	4	5	1	2	4	3	3.00	3.00	11
	21.1%	26.3%	5.3%	10.5%	21.1%	15.8%			
Echo	0	4	2	5	6	3	4.00	2.00	8
	0.0%	20.0%	10.0%	25.0%	30.0%	15.0%			
Cardiac anesthesia	1	5	2	2	5	5	4.50	3.75	4
	5.0%	25.0%	10.0%	10.0%	25.0%	25.0%			
Adult cardiac surgery (R4,5,6&7)	3	2	2	1	4	3	4.00	3.00	5
	20.0%	13.3%	13.3%	6.7%	26.7%	20.0%			
Pediatric cardiac surgery (R4,5,6&7)	0	3	0	0	6	3	5.00	3.00	1
	0.0%	25.0%	0.0%	0.0%	50.0%	25.0%			

The evaluation of the Cardiac Surgery Training Program revealed distinctive perceptions across various rotations. Cardiac Surgery itself obtained favorable ratings, boasting a median score of 4.00, indicative of a generally perceived high quality ("very good"). However, the responses exhibited some variability, as denoted by the interquartile range (IQR) of 2.50. This rotation secured a respectable rank of 7 in the overall assessment. In contrast, the General Surgery rotation garnered a median rating of 2.00, positioning it closer to an "Acceptable" perception. With a moderate IQR of 1.75, reflecting varied opinions, it obtained a lower rank of 13 within the program.

The Research rotation fell between "Acceptable" and "Good" with a median rating of 3.00. The IQR of 2.00 underlines some diversity in participant opinions, resulting in a rank of 12 within the program evaluation. The intensive care unit (ICU) rotation received a commendable median rating of 4.00, signifying a perception of "Very good." The relatively lower IQR of 1.50 suggests more consensus among participants, securing a rank of 9. Thoracic Surgery emerged as a standout rotation, achieving a high median rating of 5.00, signaling an overall perception of "Excellent". Despite a moderate IQR of 1.75, indicating varied opinions, it secured an impressive rank of 2 within the program.

Likewise, the Vascular Surgery rotation earned a notable median rating of 5.00, aligning with an "Excellent" perception. The lower IQR of 1.00 implies a more consistent consensus among participants, resulting in a rank of 3 in the program evaluation. These insights provide a nuanced understanding of the strengths and areas for potential improvement within the Cardiac Surgery Training Program. The results focus on the residents' perception of various aspects of the program. Here are the key findings.

Rotational Communication and Feedback

Participants reported a high percentage (50%) discussing rotational goals at the beginning, but a lower percentage (18%) had mid-rotation feedback and exit interviews.

Junior to Senior Transition

Only 26% of residents felt they had achieved 80% of the expected junior skills before promotion to the senior level.

Operative and Surgical Case Preparation

Participants reported discussions with primary surgeons before cases (40%) and having a plan for their specific role (45%). However, only 22% debriefed about unexpected difficulties.

Academic Performance

The majority believe the teaching is sufficient for exams and regularly attend teaching sessions.

Mentorship and Future Outlook

Satisfaction with mentors was reported by 42% of residents, and 50% felt well-secured about future job prospects.

Program data evaluation and perception

Table 3 offers a comprehensive view of participants' perspectives on diverse facets of the program. The items covered range from feedback mechanisms and goal discussions to academic preparation, mentorship, and concerns about the future. The analysis provides valuable insights into each aspect.

**Table 3:** Participants' evaluations and perceptions of various aspects of the program. SA: strongly agree, A: agree, N: neutral, D: disagree, SD: strongly disagree.

Please rate the following items	SD	D	N	A	SA	Median	IQR	Rank
My program uses resident feedback constructively	4	8	7	6	1	3.0	2.0	7
	15.4%	30.8%	26.9%	23.1%	3.8%			
I feel there is a lot of ambiguity and vagueness in the program	1	3	11	6	5	3.0	1.0	17
	3.8%	11.5%	42.3%	23.1%	19.2%			
I discuss goals and objectives with my rotational supervisor at the beginning of my rotation	4	6	2	8	6	4.0	2.3	1
	15.4%	23.1%	7.7%	30.8%	23.1%			
I usually have mid of rotation feedback session to ensure my goals and objectives has been met	4	15	2	4	1	2.0	1.0	18
	15.4%	57.7%	7.7%	15.4%	3.8%			
I usually have exist interview with my supervisor at the end my rotation	5	13	3	4	1	2.0	1.0	19
	19.2%	50.0%	11.5%	15.4%	3.8%			
I feel I have learned and achieved 80% of my proposed goals and objectives of my junior years before I am promoted to the senior years	3	5	11	5	2	3.0	2.0	8
	11.5%	19.2%	42.3%	19.2%	7.7%			
I discuss the indication of surgery and operative plan with the primary surgeon before scrubbing in the case	4	3	8	8	3	3.0	2.0	9
	15.4%	11.5%	30.8%	30.8%	11.5%			
I have clear plan of my role in each case I scrub in with primary surgeon	3	6	5	9	3	3.0	2.0	10
	11.5%	23.1%	19.2%	34.6%	11.5%			
Usually, my involvement in the surgery is very appropriate to my level of training	7	3	6	7	3	3.0	3.0	5
	26.9%	11.5%	23.1%	26.9%	11.5%			
I am allowed reasonable time and guidance to achieve my proposed task during the case	4	3	7	9	3	3.0	2.0	11
	15.4%	11.5%	26.9%	34.6%	11.5%			
I usually debrief at the end of the case with the primary surgeon in case of any difficulties or struggles during the case	4	7	9	5	1	3.0	1.3	16
	15.4%	26.9%	34.6%	19.2%	3.8%			
Teaching provided in my program is enough to prepare me to pass my written exams	3	4	9	8	2	3.0	2.0	12
	11.5%	15.4%	34.6%	30.8%	7.7%			
Teaching method used in my program is interactive and fruitful	4	2	13	4	3	3.0	1.3	15
	15.4%	7.7%	50.0%	15.4%	11.5%			
I attend all the teaching sessions provided in my program as it adds to my knowledge	0	0	10	12	4	4.0	1.0	2
	0.0%	0.0%	38.5%	46.2%	15.4%			
I attend all the teaching sessions provided in my program because it is mandatory	2	1	10	11	2	3.5	1.0	4
	7.7%	3.8%	38.5%	42.3%	7.7%			
I have an excellent mentor who guide me during my residency and advocate on my behalf	6	4	5	6	5	3.0	2.3	6
	23.1%	15.4%	19.2%	23.1%	19.2%			
I am afraid about the future after graduation from the program in terms of: (a) I feel I will not have enough knowledge of cardiac surgery	2	5	9	9	1	3.0	2.0	13
	7.7%	19.2%	34.6%	34.6%	3.8%			
I am afraid about the future after graduation from the program in terms of: (b) I feel I will not have enough surgical skills	1	2	10	7	6	3.5	1.3	3
	3.8%	7.7%	38.5%	26.9%	23.1%			
I am afraid about the future after graduation from the program in terms of: (c) I feel I will not be accepted/employed in any respectful Hospital/institution	2	6	7	7	4	3.0	2.0	14
	7.7%	23.1%	26.9%	26.9%	15.4%			

Constructive Use of Resident Feedback

Residents perceive a moderately constructive use of feedback within the program, with a median rating of 3.0, indicating a central tendency towards agreement. However, there's some variability, as reflected by the IQR of 2.0. 

Ambiguity and Vagueness in the Program

Participants express a moderate level of agreement regarding ambiguity and vagueness in the program, as evidenced by the median rating of 3.0. The narrow IQR of 1.0 suggests a more consistent perception.

Goal Discussions With Rotational Supervisor

Residents generally engage in goal discussions with their rotational supervisors, as indicated by the positive perception reflected in the median rating of 4.0. However, there's variability in experiences, as seen in the IQR of 2.3.

Mid-Rotation Feedback Sessions

Mid-rotation feedback sessions are less common, with a median rating of 2.0, suggesting a less frequent practice. The narrow IQR of 1.0 indicates a consistent perception among participants.

Exit Interviews at the End of Rotation

End-of-rotation exit interviews are infrequent, with a median rating of 2.0. The narrow IQR of 1.0 suggests a consistent perception, but the overall score is lower, indicating a less common practice.

Achievement of Junior Goals Before Promotion

On average, residents feel they have achieved a moderate level (median rating of 3.0) of proposed goals before transitioning to the senior level. The IQR of 2.0 suggests some variability in experience.

Discussion of Surgical Cases With Primary Surgeon

Residents often discuss surgical cases with primary surgeons (median rating of 3.0). The IQR of 2.0 indicates variability in experience among participants.

Clarity of Role in Surgical Cases

Residents report having a moderately clear plan for their role in surgical cases, as reflected in the median rating of 3.0. The IQR of 2.0 suggests variability in experience.

Appropriateness of Involvement in Surgery

Participants generally feel that their involvement in surgeries aligns appropriately with their training level, as indicated by the median rating of 3.0. The wide IQR of 3.0 suggests varied experiences.

Time and Guidance During Cases

Residents perceive reasonable time and guidance during cases (median rating of 3.0), with an IQR of 2.0 indicating some variability.

Debriefing After Cases

Debriefing after cases is moderately practiced, with a median rating of 3.0. The narrow IQR of 1.3 indicates a more consistent perception among participants.

Sufficiency of Teaching for Exams

Participants generally feel that the teaching in the program is sufficient for exam preparation (median rating of 3.0). The IQR of 2.0 indicates some variability in experience.

Interactive and Fruitful Teaching Methods

Teaching methods are perceived as moderately interactive and fruitful (median rating of 3.0), with a narrow IQR of 1.3 indicating consistent perception.

Attendance of Teaching Sessions for Knowledge

Residents express a high level of commitment to attending teaching sessions, with a median rating of 4.0 and a narrow IQR of 1.0 indicating a consistent perception.

Mandatory Attendance Influence

Mandatory attendance moderately influences residents (median rating of 3.5), with a narrow IQR of 1.0 indicating consistent perception.

Quality of Mentorship

Residents perceive the quality of mentorship as moderate (median rating of 3.0). The IQR indicates some variability in experience among participants.

Part 4: Satisfaction

Table 4 offers a comprehensive overview of participants' levels of satisfaction across various aspects related to their work and educational experiences. In work-related satisfaction, emotional exhaustion is apparent, with a median of 4.00, indicating participants often feel emotionally drained from their work. A similar pattern is observed for feeling used up at the end of the workday, resulting in a median of 4.50. Additionally, participants report fatigue when facing another workday, as shown by a median of 3.50.

**Table 4:** Participants' levels of satisfaction across various aspects related to their work and educational experiences. SA: strongly agree, A: agree, N: neutral, D: disagree, SD: strongly disagree.

Work and educational experience aspects	Never	A few times a year or less	Once a month or less	A few times a month	Once a week	A few times a week	Every day	Median	IQR	Rank
I feel emotionally drained from my work	3	5	2	6	1	5	4	4.00	4.00	5
	11.5%	19.2%	7.7%	23.1%	3.8%	19.2%	15.4%			
I feel used up at the end of the workday	2	1	4	6	1	4	8	4.50	4.00	4
	7.7%	3.8%	15.4%	23.1%	3.8%	15.4%	30.8%			
I feel fatigued when I get up in the morning and have to face another day on the job	1	8	4	6	2	2	3	3.50	3.00	7
	3.8%	30.8%	15.4%	23.1%	7.7%	7.7%	11.5%			
I deal very effectively with the problems of my patients	0	2	0	2	1	5	16	7.00	1.00	1
	0.0%	7.7%	0.0%	7.7%	3.8%	19.2%	61.5%			
I feel I'm positively influencing other people's lives through my work	0	2	3	3	2	6	10	6.00	3.00	2
	0.0%	7.7%	11.5%	11.5%	7.7%	23.1%	38.5%			
I feel very energetic	1	0	6	4	3	6	6	5.00	3.25	3
	3.8%	0.0%	23.1%	15.4%	11.5%	23.1%	23.1%			
I feel frustrated by my job	4	6	2	6	1	2	5	4.00	4.00	6
	15.4%	23.1%	7.7%	23.1%	3.8%	7.7%	19.2%			
	SD	D	N	A	SA	Median	IQR	Rank
Faculty and staff in my program care about my educational success	5	2	8	10	1	3.00	2.00	2
	19.2%	7.7%	30.8%	38.5%	3.8%			
I feel I am well respected and valuable person in my division	2	3	10	9	2	3.00	1.00	3
	7.7%	11.5%	38.5%	34.6%	7.7%			
I would choose the same cardiac surgery residency program again if I had the chance	4	6	6	4	6	3.00	2.25	1
	15.4%	23.1%	23.1%	15.4%	23.1%			
Overall satisfaction						4.3		

However, it is notable that participants report effective problem-solving skills in patient care, resulting in a high median of 7.00. They also express a sense of positive influence on others' lives through their work, with a median of 6.00. Energetic feelings are reported frequently, as indicated by a median of 5.00. Frustration is also evident, with participants reporting it at a median of 4.00. Also in educational satisfaction, participants perceive a high level of care from faculty and staff towards their educational success, evident in a median of 3.00. Likewise, they feel well respected and valuable within their division, with a median of 3.00.

Lastly, participants express a strong inclination to choose the same cardiac surgery residency program again if given the chance, reflected in a median of 3.00. Overall satisfaction had a median of 4.3, indicating moderate satisfaction.

## Discussion

The inception of the Cardiac Surgery Training Program in Saudi Arabia in 2011 marked the beginning of a transformative initiative in medical education [3]. As a relatively nascent program, it is poised for continuous evolution and enhancement. In our pursuit of advancing medical education research, we endeavored to pioneer an in-depth examination of the program's structural components, aiming to comprehensively evaluate its framework and gauge the overall satisfaction of participants. Since the Board of Cardiac Surgery is a relatively new training program, we sought to offer insightful information and useful data to enhance the general caliber of the local cardiac training program, since trainee productivity and satisfaction are directly correlated [7]. This study aspires to contribute valuable insights that can inform ongoing improvements and set a precedent for the critical evaluation of emerging medical training initiatives.

Examining the program's qualifications, our study included 26 participants, with 65.4% being male and 34.6% female. While positive evaluations were predominant regarding the effectiveness of the cardiac surgery rotation, a noteworthy 20.0% reported poor effectiveness in this aspect. Specific rotations, such as general surgery and the Cath lab, also exhibited effectiveness concerns ranging from 16.7% to 21.1%. These findings underscore potential areas for improvement that could adversely impact future program performance. Our study resonates with prior research revealing that a substantial 78% of Saudi surgical residents expressed dissatisfaction with the existing general surgery program [4]. Although this study was conducted in 2007-2008, indicating a need for continuous assessment and modification, it emphasizes the critical role of studying program results to guide enhancements.

Drawing from one study where 58.0% of general surgeons contemplated leaving the program due to structural issues causing severe sleep deprivation, we recognize the significance of identifying barriers affecting program structure [8]. Our study highlights the need for addressing such barriers to maximize the program's benefits for residents. In that specific study, they found that there were a lot of barriers that affected the program structure that we need to highlight to make the program more beneficial to the residents for the best outcome. In contrast, high confidence and satisfaction were found among chief residents in general surgery [9]. The discrepancy in findings could be attributed to differences in investigation focus, with our study emphasizing structural barriers. Notably, our study identified a lack of strict requirements for attendance in certain rotations, suggesting a need for increased mandatory attendance to enhance rotation effectiveness.

In our investigation of mentorship, our study revealed a noteworthy satisfaction level among participants who experienced the support of an outstanding mentor offering guidance and advocacy throughout their residency. This aligns with the results of another study where residents with mentors readily available for practical skill assistance and who seldom felt inadequately supervised during procedures reported higher satisfaction with their training, consistent with our findings [7]. Conversely, the shortage of consistent mentors among Saudi board surgical trainees was highlighted, indicating a potential source of dissatisfaction [4]. Our study indicates residents perceive an insufficiency in knowledge of cardiac surgery, potentially attributed to inadequate interviews and mid-rotation feedback. This underscores the crucial role of feedback mechanisms in knowledge and skill development.

The overall moderate level of satisfaction among residents in our study is linked to the support provided by faculty and staff for educational success. This stands in contrast to the dissatisfaction observed among cardiac surgery residents, as indicated by another investigation [10]. Our recent evaluation contributes to the evolving nature of the program, offering valuable insights for further enhancements.

Limitations

Our study, originally intended to encompass all cardiac surgery residents, faced challenges in participant recruitment, leading to a partial representation of the resident population. Excluding some residents introduces potential biases, impacting the generalizability of findings. The limited sample size may not fully capture diverse perspectives within the program. Despite these challenges, insights from included participants contribute to understanding program dynamics. For a comprehensive evaluation, it is recommended to conduct future studies with broader inclusion and larger samples.

## Conclusions

Our study provides valuable insights into the Cardiac Surgery Training Program in Saudi Arabia. While participant satisfaction varies across rotations, especially in general surgery, the findings underscore the significance of structured communication and mentorship. The study suggests crucial areas for improvement, emphasizing the need for enhanced feedback mechanisms and faculty commitment to resident education. Despite the limitations of a limited sample size, the study highlights the program's potential and the importance of ongoing assessments. Recommendations include refining attendance requirements and optimizing knowledge acquisition. Continuous research and resident-informed interventions are vital for the program's evolution and effectiveness.
